# Zinc Resistance Mechanisms of P_1B_-type ATPases in *Sinorhizobium meliloti* CCNWSX0020

**DOI:** 10.1038/srep29355

**Published:** 2016-07-05

**Authors:** Mingmei Lu, Zhefei Li, Jianqiang Liang, Yibing Wei, Christopher Rensing, Gehong Wei

**Affiliations:** 1 State Key Laboratory of Crop Stress Biology in Arid Areas, College of Life Sciences, Northwest A and F University, Yangling, Shaanxi, China; 2 College of Life Sciences, Nankai University, Tianjin, China; 3 Institute of Urban Environment, Chinese Academy of Sciences, Xiamen, China

## Abstract

The *Sinorhizobium meliloti (S. meliloti*) strain CCNWSX0020 displayed tolerance to high levels exposures of multiple metals and growth promotion of legume plants grown in metal-contaminated soil. However, the mechanism of metal-resistant strain remains unknown. We used five P_1B_-ATPases deletions by designating as ∆*copA1b*, ∆*fixI1*, ∆*copA3*, ∆*zntA* and ∆*nia*, respectively to investigate the role of P_1B_-ATPases in heavy metal resistance of *S. meliloti*. The ∆*copA1b* and ∆*zntA* mutants were sensitive to zinc (Zn), cadmium (Cd) and lead (Pb) in different degree, whereas the other mutants had no significant influence on the metal resistance. Moreover, the expression of *zntA* was induced by Zn, Cd and Pb whereas *copA1b* was induced by copper (Cu) and silver (Ag). This two deletions could led to the increased intracellular concentrations of Zn, Pb and Cd, but not of Cu. Complementation of ∆*copA1b* and ∆*zntA* mutants showed a restoration of tolerance to Zn, Cd and Pb to a certain extent. Taken together, the results suggest an important role of *copA1b* and *zntA* in Zn homeostasis and Cd and Pb detoxification in *S. meliloti* CCNWSX0020.

Heavy and transition metal homeostasis is crucial in all biological systems. Transition metals such as zinc (Zn), copper (Cu), and iron (Fe) are essential micronutrient that are required for many physiological processes but are also extremely toxic in excess. Other metals, such as cadmium (Cd), silver (Ag) and lead (Pb), are acutely toxic and represent a major threat for cell survival^1^,^2^. Organisms have evolved to contain multiple defense mechanisms to prevent overaccumulation of heavy and transition metals, such as efflux transport, intracellular sequestration, precipitation, bioadsorption and transformation, etc.^3^,^4^,^5^,^6^.

The P_1B_-type ATPase subfamily belongs to the P-type ATPases family and couples ATP hydrolysis to transition metal transport across cellular membranes. The P_1B_-type ATPases are the most widely distributed group and have the largest substrate range^7^. P_1B_-ATPases display several structural characteristics that include six to eight transmembrane helices (TMs)^8^, the signature sequence (CPC, CPH, SPC, PCP) present in the sixth TM (TM6), one or more metal binding domains in the cytoplasmic N-terminal or C-terminal region (N-MBD, C-MBD) and the catalytic phosphorylation site (DKTGT) between TM6 and TM7^9^. The P_1B_-ATPases play an essential role in transition metal homeostasis. P_1B_-ATPases have been divided into five subclasses designated P_1B-1_ to P_1B-5_ according to a combination of substrate specificity, sequence similarity and conserved metal binding residues present in transmembrane segments^9^,^10^. The metal specificity of the five ATPase subfamilies have been extensively studied and it has been shown that the P_1B-1_-ATPases transport Cu^+^ and Ag^+ ^11,12, the P_1B-2_-ATPases transport Zn^2+^, Cd^2+^ and Pb^2+ ^13,14, the P_1B-3_-ATPases are suggested to transport Cu^2+ ^15 and the P_1B-4_-ATPases transport Co^2+^, Ni^2+^ and/or Zn^2+ ^16. As to P_1B-5_ ATPase, only the *S. meliloti* Sma1163 gene encoding for a P_1B-5_-ATPase that denoted Nia was biochemically characterized indicating Ni^2+^ and Fe^2+^ are substrates of Nia^17^. Moreover, two new subtype of P_1B_-ATPases (P_1B-6_, P_1B-7_) have been recognized recently, but none of these P_1B-6_ and P_1B-7_ ATPases were biochemically examined beyond their sequence classification^18^.

Previous studies on *S. meliloti* 2011 P_1B-1_-ATPases showed that five homologous Cu^+^-ATPases exhibited functional diversity^19^. These five homologous Cu^+^-ATPases are divided into three major subgroups, including CopA1-like transporter (CopA1a and CopA1b), CopA2-like ATPase (FixI1 and FixI2) and CopA3-like ATPase (CopA3). CopA1a is a typical Cu^+^-ATPase catalyzing cytoplasmic Cu^+^ efflux to prevent overaccumulation of cytoplasmic copper. A mutation of *copA1a* resulted in a copper sensitive phenotype and an increase in cytoplasmic copper levels. CopA1b displayed 80% homology to CopA1a. However, a mutant of *copA1b* changed neither copper tolerance nor cytoplasmic copper accumulation. Rather, it changed the differentiation of the mutant strain into bacteroids, the number of viable bacteria (undifferentiated bacteria) in the *copA1b* mutant strain-induced nodules was increasing faster than differentiated bacteroids. The CopA2-like (FixI) ATPase in *S. meliloti* 2011, is encoded in an operon together with genes encoding cytochrome oxidase subunits. It has been proposed that FixI1 ATPase may be involved in respiration under microaerobiosis during symbiosis while FixI2 ATPase is required for respiration during all steps of bacterial life. CopA3 looks like a novel Cu^+^-ATPase. Mutation of *copA3* gene did not lead to sensitivity to Cu^+^ or cytoplasmic copper accumulation in the mutant strain and the gene was regulated by redox stress and was required during symbiosis. The P_1B-5_ ATPase has not been well characterized. It was shown that *S. meliloti* 2011 Nia was induced by Fe^2+^ and Ni^2+^ and a *nia* mutant accumulated nickel and iron, suggesting a possible role in Fe^2+^ and Ni^2+^ detoxification^17^.

P_1B-2_-ATPases (Zn^2+^/Cd^2+^/Pb^2+^ transporter) are less studied in rhizobia but have been well characterized in other bacteria such as ZntA in *Escherichia coli* and CadA in *Staphylococcus aureus*. ZntA is not only for Zn^2+^ efflux but also transports the non-physiological substrates Cd^2+^ and Pb^2+ ^13. The expression of the *zntA* gene is activated via the Zn^2+^-responsive transcriptional regulator (ZntR)^20^. *CadA* is known to encode a Cd^2+^ efflux ATPase which plays a role in the cadmium resistance of *S. aureu*s^21^. In *S. meliloti* 1021 the SMc04128 gene encodes a P_1B-2_-type ATPase. A transposon insertion mutant of SMc04128 showed sensitivity to high concentrations of Zn^2+^ and Cd^2+^ and slightly increased sensitivities to Cu^2+^, Pb^2+^, Ni^2+^, and Co^2+^, which indicated that SMc04128 plays a role in the defense of *S. meliloti* 1021 against these heavy metals^22^. Moreover, it has been reported that a gene named *cadA* in *Mesorhizobium metallidurans* isolated from a zinc-rich mining soil also encodes a P_IB-2_-type ATPase involved in cadmium and zinc resistance. The *cadA* gene was induced by zinc and cadmium and a *cadA*-deleted strain failed to grow at high zinc concentrations^23^.

*Sinorhizobium meliloti* CCNWSX0020 was isolated from *Medicago lupulina* growing in gold mine tailings in the northwest of China and exhibited higher tolerance towards multiple metals, such as Cu, Zn, Cd and Pb. The heavy metal transporting P_1B_-type ATPases is vital for heavy metal resistance. However, the role of P_1B_-type ATPases in *S. meliloti* CCNWSX00200 remains unknown. There are five genes encoding putative P_1B_-type ATPases on the *S. meliloti* CCNWSX0020 genome^24^. Their predicted signature transmembrane metal binding residues indicated that three genes encoded P_1B-1_-ATPase (*SM0020_05727, SM0020_05912* and *SM0020_11415*), one (*SM0020_22747*) encoded a P_1B-2_-ATPase, another (*SM0020_05862*) encoded a putative P_1B-5_-ATPase. These five P_1B_-ATPases are predicted to be mainly responsible for heavy metals homeostasis and detoxification in *S. meliloti* CCNWSX00200. To further investigate the function of these five P_1B_-ATPases we created deletions in these genes and tested different metals tolerance of these deletions. Both deletion of *SM0020_11415* (Cu^+^-ATPase) and deletion of *SM0020_22747* (Zn^2+^-ATPase) displayed sensitivity to Zn^2+^, Cd^2+^ and Pb^2+^. To test whether *SM0020_11415* and *SM0020_22747* in *S. meliloti* CCNWSX0020 have similar functions, we investigated these genes expressions in response to different levels of heavy metals exposure and their capability to complement the ∆*copA* and ∆*zntA E. coli* mutant strains. The combined results of these studies suggest that *SM0020_22747* encodes a classical Zn^2+^-ATPase which is required for efflux of Zn^2+^, Cd^2+^ and Pb^2+^, whereas *SM0020_11415* encoding a Cu^+^-ATPase surprisingly confers tolerance to Zn, Cd and Pb but not to Cu in *S. meliloti* CCNWSX00200.

## Results

### Deletion of P_1B_-type ATPases made mutant strains more sensitive to a number of heavy metals

**Figure 1:**
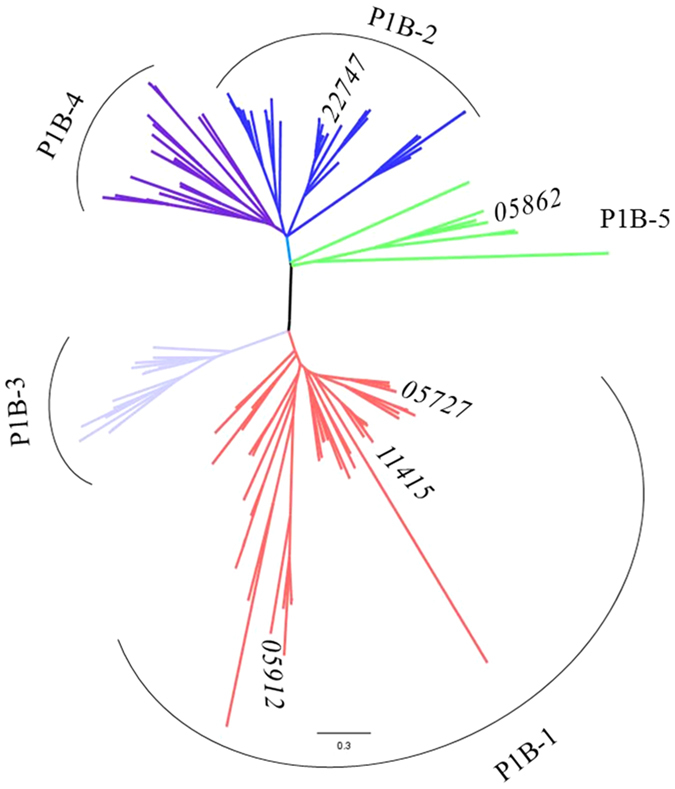
Phylogenetic analysis of P_1B_-type ATPases. Branches indicating proteins in subgroups IB-1, IB-2, IB-3, IB-4 and IB-5 are under different colors. Five P_1B_-type ATPases genes (*SM0020_05727/copA3, SM0020_05862/nia, SM0020_05912/fixI1, SM0020_11415/copA1b,* and *SM0020_22747/zntA*) in *Sinorhizobium meliloti* CCNWSX0020 are tagged in the unrooted tree.

**Figure 2:**
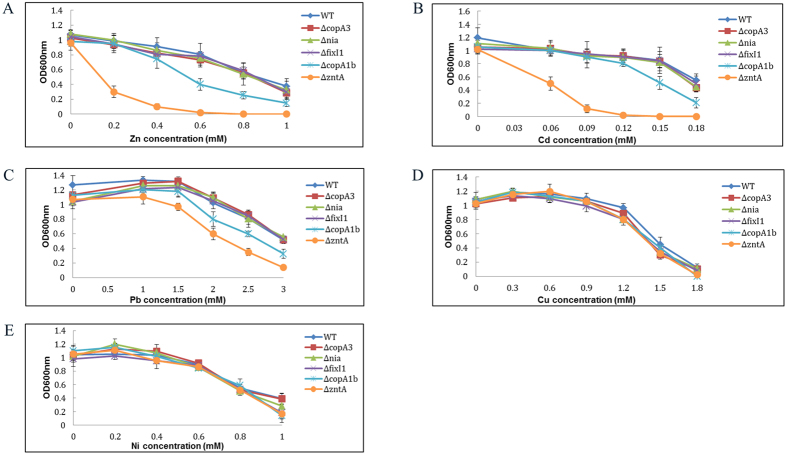
Influence of deletions in genes encoding different P_1B_-type ATPases on metal tolerance of *Sinorhizobium meliloti* CCNWSX0020. Wild type and mutant strains were grown in TY liquid medium for 48 h in the presence of increasing concentrations of zinc (**A**), cadmium (**B**), lead (**C**), copper (**D**), and nickel (**E**). Symbols represent the wild type strain(WT) and mutants Δ*copA3*, Δ*nia*, Δ*fixl1*, Δ*copA1b*, and Δ*zntA* of *S. meliloti* CCNWSX0020 (♦,■, ▲, ×, *, ●, respectively). Error bars represent standard deviations of three biological repeats.

Bioinformatics studies have shown that the genomes of many bacteria including *S. meliloti* contain a diverse array of genes encoding a number of P_1B_-ATPases. P_1B_-ATPases have been associated with the detoxification and tolerance mechanism of heavy and transition metals^25^. *S. meliloti* CCNWSX0020, could tolerate up to 1.4 mM CuSO_4_, 1.0 mM ZnSO_4_, 3.2 mM Pb(NO_3_)_2_, 0.25 mM CdSO_4_ and 1.0 mM NiSO_4_ in TY solid medium. A phylogenetic analysis of five predicted P_1B_-type ATPases from *S. meliloti* CCNWSX0020 indicated three of them were Cu^+^-ATPases, one was Zn^2+^-ATPase and the last belongs to P_1B-5_-type ATPase (Fig. 1). Furthermore, given the results of sequence homology analysis, the three Cu^+^-ATPases genes (*SM0020_05727, SM0020_05912* and *SM0020_11415*) displayed the highest similarity with the *copA3, fixI1*, and *copA1b* genes which were previously identified in *S. meliloti* 2011^19^. The *SM0020_22747* gene was 99.1% identical to the *zntA* gene of *S. meliloti* 1021 while *SM0020_05862* showed 98% similarity to the *nia* gene that encoded a nickel (Ni) and Fe transporter in *S. meliloti* 2011^17^,^22^. We therefore selected all five P_1B_-ATPases that involved in heavy metals resistance of *S. meliloti* CCNWSX0020. To test our hypotheses, the five P_1B_-ATPase deletions (∆*copA1b*, ∆*copA3*, ∆*fixI1*, ∆*zntA*, and ∆*nia*) were characterized using metal-tolerance growth assays in TY liquid medium. The wild type strain and five deletion mutants were cultured in TY supplemented with increasing concentration of CuSO_4_, ZnSO_4_, CdSO_4_, Pb(NO_3_)_2_ and NiCl_2_ (Fig. 2). The ∆*zntA* mutant showed the greatest sensitivities to 0.2 mM Zn^2+^ and 0.05 mM Cd^2+^, while ∆*copA1b* mutant was slightly more sensitive to high concentration of Zn^2+^ (0.6 mM) and Cd^2+^ (0.15 mM) (Fig. 2A,B). Both ∆*zntA* and ∆*copA1b* mutants showed sensitivity to high concentration of Pb^2+^ in different degrees and had no effect on Cu and Ni tolerance (Fig. 2C–E). The CuSO_4_, ZnSO_4_, CdSO_4_, Pb(NO_3_)_2_ and NiCl_2_ metals tolerance of the other three mutants (∆*copA3*, ∆*fixI1*, and ∆*nia*) displayed no difference to the wild type strain (Fig. 2). These results suggested CopA1b and ZntA were involved in Zn, Cd and Pb metals resistance in *S. meliloti* CCNWSX0020. To further explore the function of CopA1b and ZntA, these two zinc sensitive deletions were further studied.

### CopA1b and ZntA could play a role in zinc, cadmium and lead homeostasis

**Figure 3:**
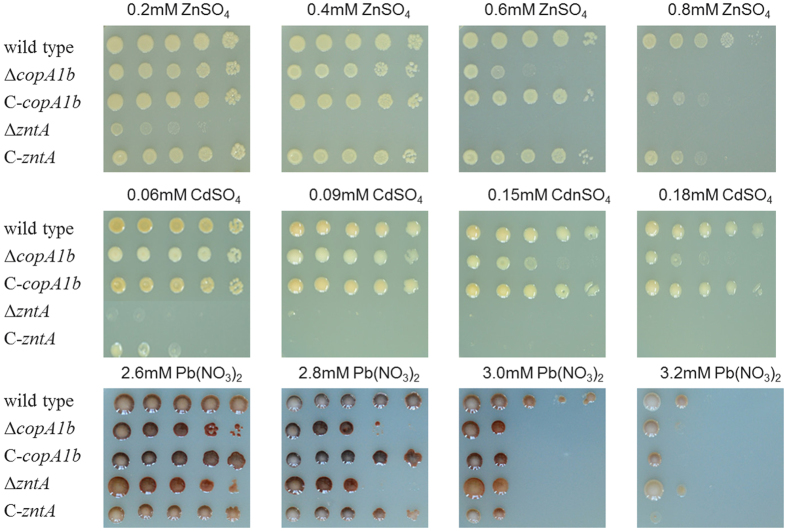
Growth in TY solid media of wild type, mutant strains (∆*copA1b* and ∆*zntA*) and complemented strains (C-*copA1b* and C-*zntA*) of *S. meliloti* CCNWSX0020. Five 10-fold dilutions were spotted from left to right, in the presence of the indicated concentrations of ZnSO_4_, CdSO_4_ and Pb(NO_3_)_2_.

**Figure 4:**
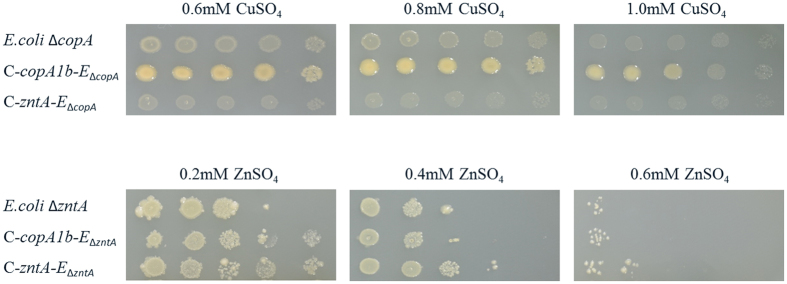
Complementation of Δ*copA E. coli* copper sensitive phenotype and Δ*zntA E. coli* zinc sensitive phenotype by heterologously expressed *S. meliloti* Cu^+^-ATPase (C-*copA1b*) and Zn^2+ −^ATPase (C-*zntA*). All strains were grown to exponential phase in LB liquid medium. Five 10-fold dilutions were carried out and spotted on the LB agar plates from left to right, in the presence of the indicated concentrations of CuSO_4_ and ZnSO_4._

The ∆*copA1b* and ∆*zntA* mutants of *S. meliloti* CNWSX0020 exhibited sensitivity to Zn^2+^, Cd^2+^ and Pb^2+^ but not to other metals, suggesting CopA1b and ZntA may play a role in Zn, Cd and Pb homeostasis in this strain. To verify the presence of the respective *copA1b* and *zntA* genes or either gene alone that was responsible for Zn, Cd and Pb resistance, the two genes were amplified and inserted into the pBBR1MCS-5 vector and transformed into the corresponding mutant and then tested for these metals tolerance. Figure 3 shows the complemented strains (C-*copA1b* and C-*zntA*) could restore Zn, Cd and Pb resistance of the mutants by 80%. These results demonstrated that the sensitivity of three metals to the mutants was due to the deletion of *copA1b* and *zntA* in *S. meliloti* CCNWSX0020. Thus we can speculate that *copA1b* and *zntA* genes are involved in Zn homeostasis and Cd or Pb detoxification. Surprisingly *zntA* encodes a Zn^2+^/Cd^2+^/Pb^2+^ transporter whereas *copA1b* is predicted to encode a Cu^+^/Ag^+^ transporter. A mutant containing a *zntA* deletion did not grow in the presence of low Zn levels as expected. In contrast, the growth of a mutant containing a *copA1b* deletion was not inhibited by high Cu levels but rather by high concentrations of Zn, Cd and Pb. To better understand the differences between the two genes in responsive to metals exposure, the capabilities of *S. meliloti* CopA1b and ZntA to complement the *E. coli* ∆*copA* and ∆*zntA* strains were tested. As expected, CopA1b could complement the Cu sensitive phenotype of *E. coli* ∆*copA* strain while ZntA could complement the Zn sensitive phenotype of *E. coli* ∆*zntA* strain. Meanwhile, CopA1b could restore Zn tolerance of an *E. coli* ∆*zntA* strain to some degree whereas ZntA failed to restore Cu tolerance of an *E. coli* ∆*copA* strain (Fig. 4). These results suggested that CopA1b and ZntA conferred resistance to Zn, Cd and Pb tolerance and CopA1b also had a capacity for Cu tolerance.

### Expression of *copA1b* and *zntA* could be induced by different types of heavy metals

**Figure 5:**
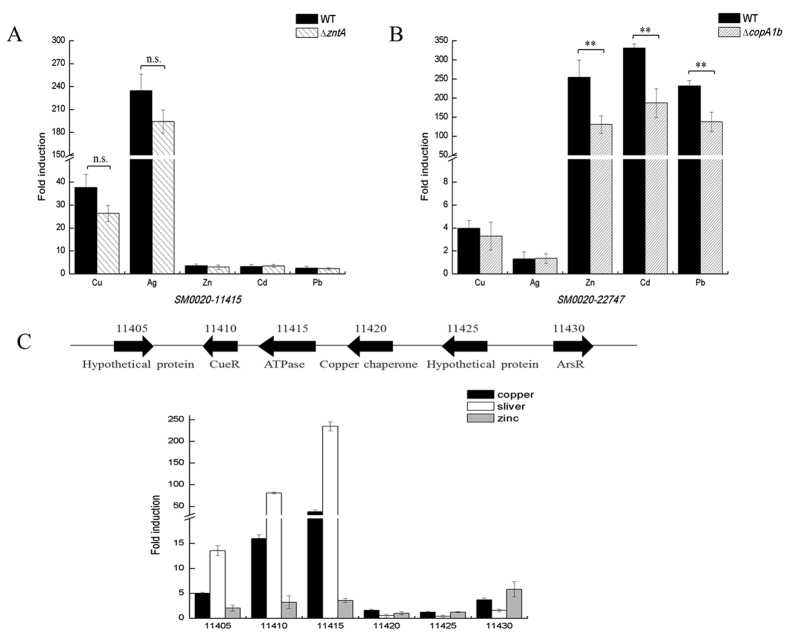
Gene expression analysis. Expression of *copA1b (SM0020_11415*) and *zntA (SM0020_22747*) under copper, silver, zinc, cadmium and lead stress (**A,B**). (**C**) Expression of genes in the vicinity of *copA1b (SM0020-11415*) under copper, silver and zinc stress. Wild type, ∆*copA1b*, and ∆*zntA* mutants of *S. meliloti* CCNWSX0020 strains at OD_600_ of 1.0 were incubated with 0.6 mM CuSO_4_, 0.05 mM AgNO_3_, 0.4 mM ZnSO_4_, 0.1 mM CdSO_4_ and 1.5 mM Pb(NO_3_)_2_ for 30 min. Samples were then processed for qPCR analysis and normalized against the ribosomal 16 S rRNA. Error bars represent standard deviations of three biological repeats. **P < 0.01.

To further investigate CopA1b and ZntA in *S. meliloti* CCNWSX0020, the gene expressions of *zntA* and *copA1b* under different metal stresses were examined using qRT- PCR. The expression profiles of *copA1b* and *zntA* genes in wild type, ∆*copA1b* and ∆*zntA* mutants in response to Cu, Ag, Zn, Cd and Pb showed some unexpected results. The expression of *copA1b* was strongly up-regulated by Ag (234-fold) and Cu (37-fold) exposure respectively (Fig. 5A), while the expression of *zntA* was significantly induced by Zn (254-fold), Cd (330-fold) and Pb (231-fold) respectively (Fig. 5B). In addition, a deletion of *copA1b* significantly inhibited *zntA* gene expression by nearly 50% (*P* < 0.01) compared to the wild type strain in response to the above three metals (Fig. 5B). These observations implied that ZntA was a typical Zn^2+^/Cd^2+^/Pb^2+^ transporter, whereas CopA1b contribute to Zn, Cd and Pb tolerance as a classical Cu^+^-ATPase but not contribute to Cu tolerance of *S. meliloti* CCNWSX0020.

Since ∆*copA1b* strain had an unexpected phenotype further studies of the genetic region in the vicinity of *copA1b* were performed. *SM0020_11410* gene locus was located directly upstream of *copA1b (SM0020_11415*) and predicted to be in an operon with *copA1b. SM0020_11410* encodes a transcriptional regulator which is highly homologous with CueR belonging to the MerR family^26^. CueR is copper sensor and induces the expression of the Cu^+^-translocating P-type ATPase CopA in response to Cu^+^, Ag^+^ or Au^+ ^27,28. The *copA1b* promoter contains a 7-7-7 bp inverted repeat (ACCTTCC-CATTATTT-GGAAGGA) between −35 and −10 similar to the *E. coli copA* promoter. This predicted gene *SM0020_11410* product might bind to the inverted repeat sequence and activate *copA1b* gene expression in the presence of Cu and Ag as a homolog of CueR. Figure 5C showed that *SM0020_11410* displayed a 15-, 130-, 4-fold induction by Cu^+^, Ag^+^ and Zn^2+^, respectively. A deletion of *SM0020_11410* led to a slight sensitivity to Cu and suppressed the *SM0020_11415* gene expression (data not shown), suggesting that *SM0020_11410* really regulated the expression of *copA1b (SM0020_11415*).

There are some other genes in the vicinity of *copA1b (SM0020_11415*) that may also be part of this operon. *SM0020_11405* and *SM0020_11425* both encode unknown proteins, *SM0020_11420* encodes a potential periplasmic copper chaperone and *SM0020_11430* encodes an ArsR family transcriptional regulator. These genes in the vicinity of *copA1b* might work together against heavy metal stress. The expression of the *SM0020_11405* (unknown protein) was inducible by Ag^+^ and Cu^+^, whereas *SM0020_11430* (ArsR family) was inducible by Cu^+^ and Zn^2+^. In addition, the gene expression level of *SM0020_11420* (copper chaperone) and *SM0020_11425* (unknown protein) was very low under Cu, Ag and Zn stress (Fig. 5C).

### Deletions of *copA1b* and *zntA* led to increased intracellular concentrations of zinc, lead and cadmium but not copper

**Figure 6:**
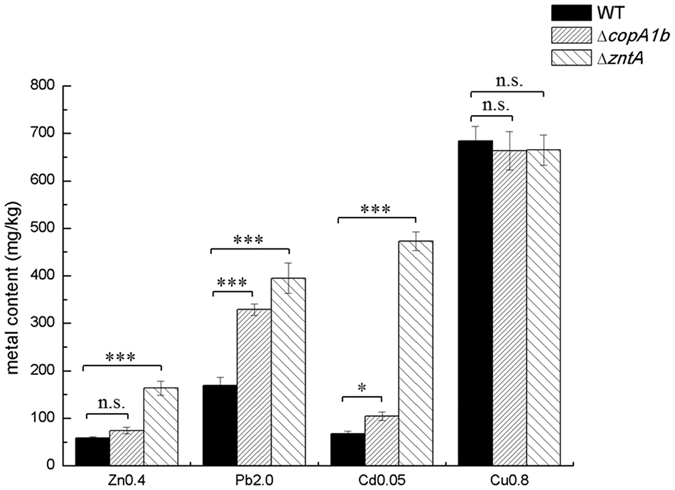
Analysis of intracellular metal concentrations in the wild type and two zinc sensitive mutants of *S. meliloti* CCNWSX0020. The wild type strain, ∆*copA1b*, and ∆*zntA* mutant strains were cultured in the presence of 0.4 mM ZnSO_4_, 2.0 mM Pb(NO_3_)_2_, 0.05 mM CdSO_4_ and 0.8 mM CuSO_4_. Cells were harvested and washed with metal-free buffer, then were dried at 65 °C and total internal metal concentrations were measured by atomic absorption spectrophotometer. Error bars represent standard deviations of three biological repeats. *P < 0.05, ***P < 0.001.

A possible explanation for the sensitivity of ∆*copA1b* and ∆*zntA* mutants toward Zn, Cd and Pb is that higher levels of these metal ions were accumulated in the mutant cells. This would imply that CopA1b and ZntA play roles in expelling these surplus metals from cytoplasm. To test this hypothesis, total internal metal content of wild type and mutant cells were measured by furnace atomic absorption spectroscopy (AAS). The cells were grown in TY medium individually supplemented with 0.4 mM ZnSO_4_, 2.0 mM Pb(NO_3_)_2_, 0.05 mM CdSO_4_ and 0.8 mM CuSO_4_. The ∆*zntA* mutant which was hypersensitive to Zn and Cd accrued significantly (*P* < 0.01) higher amounts of intracellular Zn (~3 fold), Cd (~8 fold) and Pb (~2.5 fold) compared with the WT (Fig. 6). The ∆*copA1b* mutant had a smaller but significant effect on the metal content since the loss of *copA1b* led to an increased accumulation of Zn by 1 fold, Cd by 1.5 fold (*P* < 0.05) and Pb by 2 fold (*P* < 0.01) relative to the WT (Fig. 6). The increase in the accumulation of Zn, Cd and Pb in the ∆*copA1b* and ∆*zntA* mutants suggested that CopA1b and ZntA play a role in the efflux of Zn^2+^, Cd^2+^ and Pb^2+^ ions. In addition, the accumulation of intracellular Cu was quite high in the ∆*copA1b* and ∆*zntA* mutants as well as in the WT strain (Fig. 6). This result demonstrated that CopA1b was not involved in Cu export and *S. meliloti* CCNWSX0020 accumulates high levels of intracellular Cu.

### Deletions of *copA1b* and *zntA* decreased antioxidant enzyme activity

**Figure 7:**
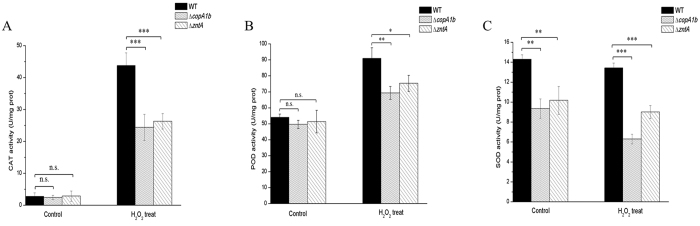
The effect of deletions of *copA1b* and *zntA* on the antioxidant activities of CAT (**A**), POD (**B**) and SOD (**C**) under H_2_O_2_ stress. The wild type strain, ∆*copA1b*, and ∆*zntA* mutant strains were cultured to exponential phase and then treated or untreated with 500 μM H_2_O_2_ for 30 min. Cells were harvested and crude bacterial lysates were subsequently prepared for spectrophotometric analyses. Error bars represent standard deviations of three biological repeats. *P < 0.05, **P < 0.01, ***P < 0.001.

P_1B_-ATPases also have a function in providing metals for assembly of periplasmic metalloproteins since some heavy and transition metals are essential component of many free-radical detoxifying enzymes, like catalase, peroxidase and superoxide dismutase^29^,^30^. So the capability of ∆*copA1b* and ∆*zntA* to cope with redox stress was tested. Although the H_2_O_2_ resistance on the agar plates of ∆*copA1b* and ∆*zntA* mutants showed no big change with *S. meliloti* wild type strain (data not shown), the CAT, POD and total SOD activities were dramatically changed. When all three strains were treated with H_2_O_2_, the CAT and POD activities increased obviously while the total SOD activity displayed no change (Fig. 7). However, compared to *S. meliloti* wild type strain, the CAT and POD activity levels of ∆*copA1b* and ∆*zntA* mutants showed apparent decline to a different degree under H_2_O_2_ treatment condition whereas the enzyme activities showed no major differences under H_2_O_2_-free condition (Fig. 7A,B). The total SOD activity of ∆*copA1b* and ∆*zntA* mutants also decreased slightly in comparison to a wild type strain whether under H_2_O_2_ treatment or not (Fig. 7C). These results suggest that deletions of CopA1b and ZntA have an influence on the activity levels of these antioxidant enzymes.

## Discussion

*Sinorhizobium meliloti* CCNWSX0020 is a multiple heavy metals resistant bacterium isolated from root nodules of *M. lupulina* growing on mine tailings in the northwest of China^31^. The genome of *S. meliloti* CCNWSX0020 has been sequenced and some copper resistance genes have been analyzed in the previous studies^24^,^32^,^33^. It has been reported that *merR* encoding an MerR family transcriptional regulator displayed significantly decreased copper resistance in a *merR*-interrupted mutant of *S. meliloti* CCNWSX0020 and the expression of two genes (*SM0020_05727* and *SM0020_05862*) encoding putative P_1B_-type ATPases were found to be down-regulated under Cu^+^/Zn^2+^/Pb^2+^/Cd^2+^ stresses in this *merR* mutant^32^. These results suggested that the P_1B_-type ATPases might be involved in heavy metal resistance of *S. meliloti* CCNWSX0020. To test this hypothesis, the five P_1B_-type ATPases in *S. meliloti* CCNWSX0020 were further studied. The phylogenetic analysis of these P_1B_-type ATPases suggested that three of them are predicted to be Cu^+^-ATPase, one to be a Zn^2+^-ATPase and the last one to be a P_1B-5_-type ATPase which may be a Ni^2+^-ATPase.

According to the analysis of deletion mutants the five genes encoding P_1B_-type ATPases obtained upon double homologous recombination, a ∆*zntA* mutant was shown to be hypersensitive to low concentrations of Zn and Cd compared to other mutants and the wild type, indicating the vital role of ZntA in Zn and Cd resistance in *S. meliloti* CCNWSX0020. As pointed out in previous studies, ZntA in *S. meliloti* CCNWSX0020 contains a conserved phosphorylation motif (DKTGT), a CXXC (CASC) motif in N terminal and typical conserved residues of P_1B-2_ ATPases in trans-membrane^8^ helices including a CPC motif in TM6, a T(X)_5_QN(X)_7_K motif in TM7 and a DXG(X)_7_N motif in TM8 (see Supplementary Fig. S1)^9^,^34^. The well-characterized ZntA from *E. coli* mediates the efflux of Zn^2+^, Cd^2+^ and Pb^2+ ^13. Amino acid alignment of ZntA from *S. meliloti* CCNWSX0020 showed 98.04% identity to the SMc04128-encoded P_1B-2_-type ATPase in *S. meliloti* 1021 which plays a crucial role in the defense of *S. meliloti* against high concentrations of Zn and Cd^22^. The *S. meliloti* CCNWSX0020 ∆*zntA* mutant is highly sensitive to low concentration of Zn and Cd but only slightly sensitive to high concentration of Pb, suggesting that ZntA in *S. meliloti* CCNWSX0020 is essential to the resistance against these metal ions. RT-PCR showed that the expression of *zntA* in *S. meliloti* CCNWSX0020 was induced by heavy metals with the following order of effectiveness: Cd^2+^ > Zn^2+^ > Pb^2+^. In addition, a *S. meliloti* CCNWSX0020 ∆*zntA* mutant displayed an increased intracellular accumulation of Zn, Pb and Cd. These results strongly suggest ZntA in *S. meliloti* CCNWSX0020 to be a typical Zn^2+^-ATPase having a crucial role in the efflux of Zn, Cd and Pb.

Previous studies have reported the presence of five Cu^+^-ATPase genes on the *S. meliloti* 2011 genome and analyzed the functional diversity of these five homologous Cu^+^-ATPases^19^. The authors divided these five Cu^+^-ATPases into three subgroups including CopA1-like ATPases (CopA1a and CopA1b), CopA2-like ATPases (FixI1 and FixI2) and CopA3-like ATPases. Based on sequence alignment, three genes (*SM0020_05727, SM0020_05912* and *SM0020_11415*) encoding Cu^+^-ATPases on the *S. meliloti* CCNWSX0020 genome were very similar to CopA3, FixI1 and CopA1b, respectively. The deletions of putative Cu^+^-ATPases (∆*copA1b*, ∆*copA3* and ∆*fixI1*) had no effect on Cu resistance in agreement with results obtained from mutants of the genes encoding homologous Cu^+^-ATPases in *S. meliloti* 2011. However, one important difference from *S. meliloti* 2011 was that a ∆*copA1b* deletion in *S. meliloti* CCNWSX0020 displayed sensitivity to high concentrations of Zn, Cd and Pb. In addition, we could not identify a typical Cu^+^-translocating P_1B_-ATPase such as CopA1a in *S. meliloti* 2011 on the genome of *S. meliloti* CCNWSX0020. Moreover, cells of *S. meliloti* CCNWSX0020 accumulated quite high amounts of Cu in both the wild type strain and the different mutant strains. This could indicate that copper resistance in *S. meliloti* CCNWSX0020 is not due to efflux but rather increased copper binding in cells most likely in the periplasm.

Based on sequence alignment, *S. meliloti* CCNWSX0020 CopA1b was predicted to be a Cu^+^-translocating P_IB_-ATPases and CueR located upstream of *copA1b* was responsible for Cu and Ag-dependent induction of *copA1b* expression. Moreover, *copA1b* could confer copper tolerance to a copper sensitive *E. coli* ∆*copA* strain (Fig. 4). These results suggested that CopA1b itself has a capability for copper tolerance and/or efflux. However, ∆*copA1b* mutant in *S. meliloti* CCNWSX0020 did not lead to copper sensitivity or increased copper accumulation. Perhaps the lack of copper sensitive phenotype in the ∆*copA1b* mutant was masked by functional redundancy with other copper transporters or copper resistance determinants. Based on a previous study in *S. meliloti* CCNWSX0020, some genes responsible for copper homeostasis could be identified. These genes include *lpxXL (SM0020_18047*) encoding the LpxXL C-28 acyltransferase, *omp (SM0020_18792*) encoding a hypothetical outer membrane protein, *cueO (SM0020_18797*) encoding a periplasmic multicopper oxidase, and *merR (SM0020_29390*) encoding a regulatory activator^32^. Therefore, we compared expression of these four genes with expression of the genes encoding these three Cu^+^-ATPase (*SM20020*_*05727*/*copA3, SM0020*_*05912*/*fixI1, SM0020*_*11415*/*copA1b*) under Cu stress. The data showed that *omp* and *cueO* were highly induced by Cu to 646- and 243-fold, respectively (see Supplementary Fig. S2). Previous study showed that the *omp* mutant was hypersensitive to Cu^33^ and our data showed *omp* was highly induced by Cu, revealing Omp to play a crucial role in the copper resistance mechanism of *S. meliloti* CCNWSX0020. In addition, the capability of *S. meliloti* CopA3 to complement the *E. coli* ∆*copA* strain was also tested. The result showed that the CopA3 did not restore copper tolerance of *E. coli* ∆*copA*, indicating there is no functional redundancy of CopA3 with CopA1b (data not shown).

The *∆copA1b* mutant was sensitive to Zn, Cd and Pb, and led to increased intracellular Zn, Cd and Pb concentrations, demonstrating that although *copA1b* in *S. meliloti* CCNWSX0020 is predicted to encode a Cu^+^-ATPase, it is involved in Zn, Cd and Pb homeostasis. Previous studies have reported that P_1B-_ATPases have a high specificity for substrate they transport, for example Cu^+^-ATPase transports monovalent heavy metal ions (Cu^+^, Ag^+^) and Zn^2+^-ATPase transports divalent heavy metal ions (Zn^2+^, Cd^2+^)^9^,^10^. Sequence comparisons and functional characterization have underlined the importance of the difference between Cu^+^-ATPases and Zn^2+^-ATPases in the presence of unique and conserved trans-membrane amino acid residues which could contribute to substrate specificity, such as Tyr/Asn of TM7, Pro/Met/Ser of TM8 in Cu^+^-ATPase while Leu/Lys of TM7, and Asp/Val/Ala of TM8 in Zn^2+^-ATPase^34^,^35^,^36^. Furthermore, in agreement with most Cu^+^-ATPases, CopA1b in *S. meliloti* CCNWSX0020 contains two CXXC(CASC) motifs in the N terminal and typical conserved residues of P_1B-1_ ATPases in trans-membrane helices including a CPC motif in TM6, a YN(X)_4_P motif in TM7, a MXXSS motif in TM8 (see Supplementary Fig. S1)^9^,^34^. It is probable that the presence of the same N-terminal CASC motif in CopA1b and ZntA in *S. meliloti* CCNWSX0020 could bind Zn^2+^/Cd^2+^/Pb^2+^ ions. However, the function of the N-terminal CXXC motif in P_1B_-type ATPase remains controversial, as two N-terminal CXXC motifs in *E. coli* CopA have distinct functions (the MBD1 functions as metallochaperones and the MBD2 has a regulatory role in CopA activity)^37^ while CXXC motif in *Streptococcus pneumoniae* CopA is able to bind a dicopper center and might be responsible for delivery of Cu^+^ to the TM metal-binding sites^38^. To study the function of N-terminal CXXC motif of CopA1b and ZntA in *S. meliloti* CCNWSX0020, we constructed these two N-terminal deletions. The results showed that the *zntA* N-terminal deletion was slightly sensitive to high zinc concentration while *copA1b* N-terminal deletion did not affect copper and zinc tolerance in *S. meliloti*, and that neither N-terminal deletions of CopA1b nor ZntA could restore copper or zinc tolerance of *E. coli* ∆*copA* and ∆*zntA* mutant trains (data not shown). These indicate that the presence of N-terminal domain of CopA1b and ZntA is essential for their complete transportation function. In addition, the *S. meliloti* CopA1b confers a slight increase in zinc tolerance of *E. coli* ∆*zntA* mutant (Fig. 4). Therefore, it is very possible that the similar N-terminal domain of CopA1b to ZntA could bind Zn^2+^/Cd^2+^/Pb^2+^ ions but these metal ions binding by CopA1b N-terminal domain is not essential for cellular Zn^2+^/Cd^2+^/Pb^2+^ resistance, just played a primary role in cytoplasmic Zn^2+^/Cd^2+^/Pb^2+^ sequestration or delivery to the transmembrane site of CopA1b for cellular efflux. Notably, similar to *copA1b*, SMc04128 gene in *S. meliloti* 1021 encoded a Zn^2+^- ATPase and transposon insertion mutant of SMc04128 was not only highly sensitive to Zn^2+^ and Cd^2+^ but also slightly increased sensitivities to Cu^2+^, Pb^2+^, Ni^2+^ and Co^2+ ^22. This phenomenon has attracted our attention and it is likely that Cu^+^-ATPase and Zn^2+^-ATPase in *S. meliloti* might not have strict specificity for the heavy and transition metal ions they transport.

Moreover, P_1B-1_-ATPases were also shown to be involved in protein maturation and delivering metal cofactors into metalloenzymes^39^,^40^. Previous work demonstrated that both functions are important for bacterial virulence^25^,^41^. A novel host immune defense against bacterial invaders was identified and involved intoxication by transition metals (such as copper and zinc) in the phagosome to kill bacteria^42^,^43^,^44^. This mechanism could also be identified in response to protozoa^45^. P_1B_-ATPases in bacteria are required for transition metal efflux and assembly of metalloproteins which are essential for bacterial survival in extreme oxidative environments^46^. So we speculate that although CopA1b in *S. meliloti* CCNWSX0020 is not responsible for copper tolerance, it may incorporate copper into metalloenzymes (such as Cu/Zn-SOD) that protect against metal or oxidative stress. In line with our speculation, the deletion of *copA1b* led to a decrease in CAT, POD and total SOD activity level (Fig. 7), so a *copA1b* deletion could result in sensitivity to high concentration of Zn, since ∆*copA1b* mutant might lack enough capability to cope with the oxidative stress caused by high Zn. However, Cu and Zn usually served as cofactor for Cu/Zn-SOD not for CAT and POD, but the deletions of *copA1b* and *zntA* also decreased the CAT and POD activity levels. It is speculated that the reduced CAT and POD activity might result from the periplasmic metal disturbances caused by deletions of *copA1b* and *zntA*.

In conclusion, in S. *meliloti* CCNWSX0020 ZntA is a typical Zn^2+^-ATPase playing a crucial role in the efflux of Zn, Cd, and Pb while *copA1b* encoding a Cu^+^-ATPase is involved in tolerance to Zn, Cd, and Pb. Moreover, both of CopA1b and ZntA are potentially being involved in assembly and maturation of metalloenzymes crucial for tolerance to heavy metal and oxidative stress.

## Materials and Methods

### Bacterial strains, plasmids and culture conditions

All bacterial strains and plasmids used in this study are listed in Table 1. *Escherichia coli* strains (*E. coli* DH5α, *E. coli* GR16 and *E. coli* RW3110) were grown in Luria-Bertani (LB) medium at 37 °C. *Sinorhizobium meliloti* CCNWSX0020 was grown at 28 °C in TY medium (5 g tryptone, 3 g yeast extract, and 0.7 g CaCl_2_**·**2 H_2_O per liter)^47^. Media were supplemented with the following antibiotics as required: 100 μg/mL ampicillin (Amp), 50 μg/mL kanamycin (Km), 100 μg/mL gentamicin (Gm) (Table 1).

### Bioinformatic analysis

The known P_1B_-ATPase protein sequences of most bacterial genomes used in our study were obtained from UniProtKB (http//www.uniprot.org/uniprot/)^48^. The whole set of bacterial P_1B_-ATPase sequences were aligned using ClustalW2^49^ and the phylogenetic tree visualized with FigTree (http//tree.bio.ed.ac.uk/software/figtree/). The neighboring genes of the genes encoding P_1B_-ATPases were obtained from the draft genome sequence of *S. meliloti* CCNWSX0020 which had been reported with the accession number AGVV00000000.1 in GenBank^24^.

### Generation of deletion mutants in genes encoding P_1B_-type ATPases

An in-frame, tagged P_1B_-ATPase deletion mutant of *S. meliloti* CCNWSX0020 was constructed by a method involving crossover PCR^50^ and the suicide vector pK18mobsacB, which cannot replicate in *S. meliloti* CCNWSX0020^51^. The total genomic DNA of *S. meliloti* CCNWSX0020 was extracted according to the protocol of Wilson and Carson^52^. The plasmid pK18mobsacB-∆*copA1b* was used to construct the *S. meliloti* CCNWSX0020 *copA1b* deletion mutant. A 683 bp upstream and a 655 bp downstream fragment of *copA1b* were amplified using primer pairs copA1b-F1/copA1b-R1 and copA1b-F2/copA1b-R2, respectively. The upstream and downstream PCR products were ligated by crossover PCR with primer pairs copA1b-F1/copA1b-R2. The resulting fragment was cloned into pMD18-T vector (TaKaRa) that introduces *Xba*I/*Hind*III sites generating vector pMD18-∆*copA1b*. The plasmid pMD18-∆*copA1b* was digested with *Xba*I and *Hind*III and the ∆*copA1b* fragment was inserted into the *Xba*I/*Hind*III site of the suicide vector pK18mobsacB to produce pK18-∆*copA1b*. The plasmid pK18-∆*fixI1*, pK18-∆*copA3*, pK18-∆*zntA*, pK18-∆*Nia* were constructed in a similar manner and all primers used in this study are listed in Table S1.

The deletion was obtained through double homologous recombination. In the first step, the generated plasmid pK18-∆*copA1b* was transferred into *S. meliloti* CCNWSX0020 by triparental mating which included *S. meliloti* CCNWSX0020 (Amp^r^) as the recipient, *E. coli* DH5α cells containing pK18-∆*copA1b* (Km^r^) as the donor, and *E. coli* DH5α cells containing pRK2013 as helper cells. The selective SM agar medium (0.2 g MgSO_4_**·**7 H_2_O, 0.1 g CaCl_2_, 0.5 g KNO_3_, 0.5 g K_2_HPO_4_, 0.1 g NaCl, 10 g mannitol, 75 mg pantothenic acid, 75 mg biotin, 75 mg thiamine, and 15 g agar per liter)^53^ with kanamycin and ampicillin was used for screening the first recombination events. Since pK18-∆*copA1b* is unable to replicate in *S. meliloti* CCNWSX0020, kanamycin-resistant clones should have integrated the plasmid into the chromosome by homologous recombination via one of the *copA1b* flanking regions. In addition *S. meliloti* CCNWSX0020 contains ampicillin resistance whereas *E. coli* could not grow in SM medium. In the second step to select for a second recombination event, clones resistant to both kanamycin and ampicillin were grown in TY liquid medium with ampicillin for 24 h and plated onto TY plates containing 10% (w/v) sucrose. As 10% sucrose was lethal to single crossover clone, sucrose-resistant clones must be the wild type strain or the deletion mutant by a second crossover event. Double crossover recombinants were confirmed by PCR using copA1b-F1 and copA1b-R2 as primers. Finally, the correct PCR product was sequenced.

Other tagged P_1B_-ATPase deletion mutants of *S. meliloti* CCNWSX0020 were constructed in the similar manner as described above for the *copA1b* deletion mutant. The resulting strains were designated as ∆*copA1b*, ∆*fixI1*, ∆*copA3*, ∆*zntA* and ∆*nia* (Table 1).

### Heavy and transition metal sensitivity assays of the five mutants

*Sinorhizobium meliloti* CCNWSX0020 and each of the five deletion mutants were grown to midexponential phase in TY liquid medium at 28 °C with shaking at 150 rpm and cell suspensions were prepared at the same OD_600_ of 1.0 (optical density at 600 nm). Then 1% of the cell suspensions were added to fresh TY medium supplemented with different concentration of CuSO_4_, ZnSO_4_, CdSO_4_, NiCl_2_, and Pb(NO_3_)_2_. The cells were again incubated with shaking at 150 rpm for 48 h, and growth was monitored at OD_600_. The data were shown as the means of biological triplicates ± SD.

### Complementation of ∆*copA1b* and ∆*zntA* mutants

To complement the *copA1b* mutant, the entire *copA1b* gene including the regulatory region was amplified with primers C-copA1b-F/C-copA1b-R using *S. meliloti* CCNWSX0020 genomic DNA as template. The PCR product was digested with *Sma*I/*Xba*I and inserted into broad-range plasmid pBBR1MCS-5 to generate pBBR-*copA1b*. The sequence of this construct to be used in complementation was verified by automated DNA sequencing, transformed into *E. coli* donor strain DH5α, and delivered into the ∆*copA1b* mutant via triparental conjugation. Single clones carrying pBBR-*copA1b* were selected on TY plates containing gentamicin. The presence of the *copA1b* gene in the mutant strain was confirmed by PCR. The complementation of a *zntA* deletion was performed in a similar fashion.

Metal sensitivity assays of *S. meliloti* CCNWSX0020 wild type, ∆*copA1b* mutant and ∆*zntA* mutant and the corresponding complementations (C-*copA1b* and C-*zntA*) were performed on TY solid medium with different concentrations of CuSO_4_, ZnSO_4_, CdSO_4_ and Pb(NO_3_)_2_. Cells were grown to exponential phase in TY liquid medium and then diluted to an OD_600_ of 0.1. Four 10-fold dilutions were carried out and spotted on the agar medium. Each experiment was repeated three times.

### Heterologous expression of *S. meliloti copA1b* and *zntA* in *E. coli* ∆*copA* and ∆*zntA* strains

Complementation plamids pBBR-*copA1b* and pBBR-*zntA* were transformed into *E. coli* ∆*copA* strain and *E. coli* ∆*zntA* strain via triparental conjugation, respectively. Single clones carrying pBBR-*copA1b* or pBBR-*zntA* were selected on LB plates containing gentamicin. The presence of the *copA1b* or *zntA* gene in the *E. coli* ∆*copA* and ∆*zntA* strains was confirmed by PCR.

Copper and zinc sensitivity assays of *E. coli* ∆*copA* and ∆*zntA* strains and the corresponding complementation (C-*copA1b-E*_∆*copA*_, C-*copA1b-E*_∆*zntA*_, C-*zntA-E*_∆*copA*_ and C-*zntA-E*_∆*zntA*_) were performed on LB solid medium. Cells were grown to exponential phase at 37 °C in LB liquid medium and then diluted to an OD_600_ of 0.1. Five 10-fold dilutions were carried out and spotted on the agar medium with indicated concentrations of CuSO_4_ and ZnSO_4_. Each experiment was repeated three times.

### Heavy and transition metal accumulation assay

*Sinorhizobium meliloti* CCNWSX0020 wild type and mutant strains were grown at 28 °C in TY liquid medium until cells reached exponential phase at the same OD_600_ of 0.8. Then cells were incubated for another 24 h with shaking at 150 rpm after TY medium had been supplemented with 0.8 mM CuSO_4_, 0.4 mM ZnSO_4_, 0.05 mM CdSO_4_ and 2.0 mM Pb(NO_3_)_2_. Then cells were harvested by centrifugation at 8000 g for 10 min. The intracellular accumulated heavy and transition metals were measured by furnace atomic absorption spectroscopy (Varian SpectrAA 880/GTA 100) as described previously^31^. The data were shown as the means of biological triplicates ± SD.

### H_2_O_2_ sensitivity test and antioxidant enzyme activity assay

*Sinorhizobium meliloti* CCNWSX0020 wild type strain, ∆*copA1b* and ∆*zntA* mutants were grown to exponential phase in TY liquid medium and then diluted to an OD_600_ of 0.1. Four 10-fold dilutions were carried out and spotted on the agar medium with different concentration of H_2_O_2_ for H_2_O_2_ sensitivity test.

The exponential phase cells grown in TY liquid medium were treated or untreated with 500 μM H_2_O_2_ for 30 min. Then cells were harvested by centrifugation at 8000 g for 2 min and resuspended with enzyme extracting solution. Cell suspensions were lysed by ultrasonic disruption, followed by centrifugation at 8000 g for 10 min. Clear lysates were used for total protein determination and catalase, peroxidase and superoxide dismutase activity assay. Protein concentrations were determined by using the Bradford Bio-Rad protein assay. The catalase (CAT) activity was assayed by measurement of the degradation of H_2_O_2_ at a wavelength of 240 nm according to the method of Aebi^54^. The peroxidase (POD) activity was determined spectrophotometrically at 470 nm by the method of Hammerschmidt, Nuckles and Kuc^55^. The superoxide dismutase (SOD) activity was assayed by measuring the enzyme’s ability to inhibit the photochemical reduction of nitroblue tetrazolium (NBT) as described previously by Beauchamp and Fridovich^56^. The data were shown as the means of biological triplicates ± SD.

### Real-time qRT-PCR analysis

*Sinorhizobium meliloti* CCNWSX0020 and two zinc-sensitive mutants grown to exponential phase in TY liquid medium were supplemented with 0.6 mM CuSO_4_, 0.05 mM AgNO_3_, 0.4 mM ZnSO_4_, 0.1 mM CdSO_4_ and 1.5 mM Pb(NO_3_)_2_ and incubated for 30 min at 28 °C. Then cells were harvested and total RNA was extracted. Procedures including DNA elimination, cDNA synthesis and quantitative RT-PCR were performed as described previously^32^. All these assays were performed in triplicate. Primer pairs used to monitor transcription of genes are listed in Table S1. To standardize results, 16S rRNA was used as an internal standard and the relative levels of transcription were calculated using the 2^−∆∆Ct^ method^57^.

### Statistical analyses

Statistical analyzes were carried out using SPSS 19.0 software (SPSS 208 Inc., Chicago, IL, USA). Paired two-tailed Student’s t-test was performed to determine significant differences among the different experimental treatments. All of the data was analyzed using the Origin Pro v8.0 (Origin Lab, Hampton, USA) to create the figures.

## Additional Information

**How to cite this article**: Lu, M. *et al*. Zinc Resistance Mechanisms of P1B-type ATPases in *Sinorhizobium meliloti* CCNWSX0020. *Sci. Rep.* **6**, 29355; doi: 10.1038/srep29355 (2016).

## Supplementary Material

Supplementary Information

## Figures and Tables

**Table 1:** Bacterial strains and plasmids used in this study.

Strains or plasmids	Relevant characteristics	Source or reference
Strains		
* S. meliloti* CNWSX0020	Wild-type strain, Nod^+^ on Medicago lupulina, Amp^r^	This work
* *Δ*copA1b*	*copA1b* deleted in *S. meliloti* CNWSX0020	This work
* *Δ*copA3*	*copA3* deleted in *S. meliloti* CNWSX0020	This work
* *Δ*fixI1*	*fixI1* deleted in *S. meliloti* CNWSX0020	This work
* *Δ*zntA*	*zntA* deleted in *S. meliloti* CNWSX0020	This work
* *Δ*nia*	*nia* deleted in *S. meliloti* CNWSX0020	This work
* *Δ*cueR*	*cueR* deleted in *S. meliloti* CNWSX0020	This work
* *ΔN-*copA1b*	N-terminal domain of CopA1b deleted in *S. meliloti* CNWSX0020	This work
* *ΔN-*zntA*	N-terminal domain of ZntA deleted in *S. meliloti* CNWSX0020	This work
* E. coli* DH5α	*lacZ*ΔM15 *recA1 gyrA96 hsdR17*	58
* E. coli* GR16	Copper sensitive *E. coli* W3110; ∆*copA*::Km, ∆*cueO*::Cm, ∆*cusA*::cm	59
* E. coli* RW3110	Zinc sensitive *E. coli* W3110; ∆*zntA*::Km	14
Plasmids		
* *pK18mobsacB	Suicide vector derived from plamid pK18, Mob^+^ *sacB* Km^r^	51
* *pBBR1MCS-5	Broad-host-range cloning vector, Gm^r^	60
* *pRK2013	Broad-host-range helper vector, Tra^+^ Km^r^	University of York, UK, Tanya Soule
* *pMD18-T easy	Cloning and sequencing vector, Amp^r^	TaKaRa
