# Supplementary material for: Zinc Resistance Mechanisms of P1B-type ATPases in Sinorhizobium meliloti CCNWSX0020
Source: Sci Rep. 2016 Jul 5;6:29355. doi: 10.1038/srep29355 (PMC4932525; doi:10.1038/srep29355)
Supplement: Supplementary Information [file srep29355-s1.pdf]

Supplementary materials for

Zinc Resistance Mechanisms of P_{1B}-type ATPases in *Sinorhizobium meliloti* CCNWSX0020

Mingmei Lu[a], Zhefei Li[a], Jianqiang Liang[a],Yibing Wei[b], Christopher Rensing[c], Gehong Wei[a]*

Supplementary for Table. S1 Primers used in this study

Name	Sequence	Use
copA1b-F1	GCGAACGCCCAGAACAGA	To generate Δ*copA1b* deleted fragment
copA1b-R1	GGCTATTCGGTCGTTGCGACGCTTTCCGAGCACCC	
copA1b-F2	GGGTGCTCGGAAAGCGTCGCAACGACCGAATAGCC	
copA1b-R2	GTCCGCGAGTGTCAACCTG	
fixI1-F1	AAGGGAACTGCCGAAAGAGG	To generate *ΔfixI1* deleted fragment
fixI1-R1	ATCAGCGGTCTCGCACGCCTTGGATGCTGGTGGAGGGA	
fixI1-F2	TCCCTCCACCAGCATCCAAGGCGTGCGAGACCGCTGAT	
fixI1-R2	CATAGTTGCCGACGGAGGG	
copA3-F1	CAGTTCGCAACAACACGCAGTC	To generate Δ*copA3* deleted fragment
copA3-R1	ATCCGTGTACTCCTCAATCTGACATGGGGCAGGTGTAGATCACG	
copA3-F2	CGTGATCTACACCTGCCCCATGTCAGATTGAGGAGTACACGGAT	
copA3-R2	CCTTAAACATGATCGTAGGGTG	
zntA-F1	GCCCTGAGCCTGCTTACGA	To generate Δ*zntA* deleted fragment
zntA-R1	ATGGTCGTCTTCCTCTTCCTGATGCCGACGGAGCGTTTCAT	
zntA-F2	ATGAAACGCTCCGTCGGCATCAGGAAGAGGAAGACGACCAT	
zntA-R2	CGGAAGTCTGACCTGCACATTTT	
nia-F1	GCAAATGGGGTATGGGCAAGAT	To generate Δ*nia* deleted fragment
nia-R1	CGTTGTCGCAGCAGAGTATCCATACAGCGATGAGGGCAAGGAGA	
nia-F2	TCTCCTTGCCCTCATCGCTGTATGGATACTCTGCTGCGACAACG	
nia-R2	CCCTCGACCATTACTGGGCTCT	
cueR-F1	TGTGACGAGAAGAACCAAGAGTTT	To generate Δ*cueR* deleted fragment
cueR-R1	CTCCAAAGCCAGCGGCGTCTCCGCTGAAATAGCGTTACTGAGACT	
cueR-F2	AGTCTCAGTAACGCTATTTCAGCGGAGACGCCGCTGGCTTTGGAG	
cueR-R2	TTGCCGGAAGGCAAGGTCGAG	
N-copA1b-F1	TTCTGGACGGGAAGGGGT	To generate *copA1b* N-terminal deleted fragment
N-copA1b-R1	GCTCCGCTTCCGGTTTCCTCGGCAGGAGACGAGGACC	
N-copA1b-F2	GGTCCTCGTCTCCTGCCGAGGAAACCGGAAGCGGAGC	
N-copA1b-R2	CCAACAAGGCGGAGGAGA	
N-zntA-F1	GCGTGTAATAGCGGGAGA	To generate *zntA* N-terminal deleted fragment
N-zntA-R1	TCGGGAAACGAGATTTCGCCTTGACGTTGCGGCGGA	
N-zntA-F2	TCCGCCGCAACGTCAAGGCGAAATCTCGTTTCCCGA	
N-zntA-R2	CGACGTCCACGTCTCCTA	
C-copA1b-F	GCTGGCTTTGGAGGCTTC	To generate *copA1b* full-length fragment
C-copA1b-R	CCACTACGATCTTGTGCG	
C-zntA-F	GGGAGGGGTCTTGTCAGCG	To generate *zntA* full-length fragment
C-zntA-R	TAGGGCGTAGTCGGGGTT	
copA1b-F	CCAGGCGGCAAAAGTCAG	qPCR of *copA1b*

copA1b-R	TTCAGGGCCACCAAGGTC	
zntA-F<br>zntA-R	ACGGTTCCGGCAAAGACA<br>CGGCAGGACTGTGGAGGT	qPCR of *zntA*
omp-F<br>omp-R	GTAGCGGCTTGGGAAAC<br>AGTTCGGCGTAGAAGACAT	qPCR of *omp*
cueO-F<br>cueO-R	TCTCGGCGGACGATTAC<br>CCCGTGTTTGGAGTGCTT	qPCR of *cueO*
lpxXL-F<br>lpxXL-R	GGGAACGGCAAGACCGAGTG<br>CTGGATGCTGGTGGAGGGAGA	qPCR of *lpxXL*
merR-F<br>merR-R	GTTTCACGCTTGAGGAGAT<br>AATACGGATGTTCTCAGTTTG	qPCR of *merR*
11405-F<br>11405-R	GGAGAGGGCAGGCGAATG<br>CAGGAAGGACCAGAGCGG	qPCR of 11405
11410-F<br>11410-R	CGGCTATCTTGCGTTCCA<br>TTATCAGTCCCGCCCTTC	qPCR of 11410
11420-F<br>11420-R	AAAGGCAGCGAGACCAAT<br>GCAGCGTCCGAAAATAAA	qPCR of 11420
11425-F<br>11425-R	GTCTGCTCGCCTGACTGC<br>CGCTGGAAACCGAAAACA	qPCR of 11425
11430-F<br>11430-R	GGGTGCGATAGCAGAAAA<br>AGCTGGCGCTGTAGACGA	qPCR of 11430
16S-F<br>16S-R	GAACCTTACCAGCCCTTG<br>CATCCCCACCTTCCTCTC	qPCR of 16S rRNA
P1<br>P6	AGAGTTTGATCCTGGCTCAGAACGAACGCT<br>TACGGCTACCTTGTTACGACTTCACCCC	To generate 16S rRNA

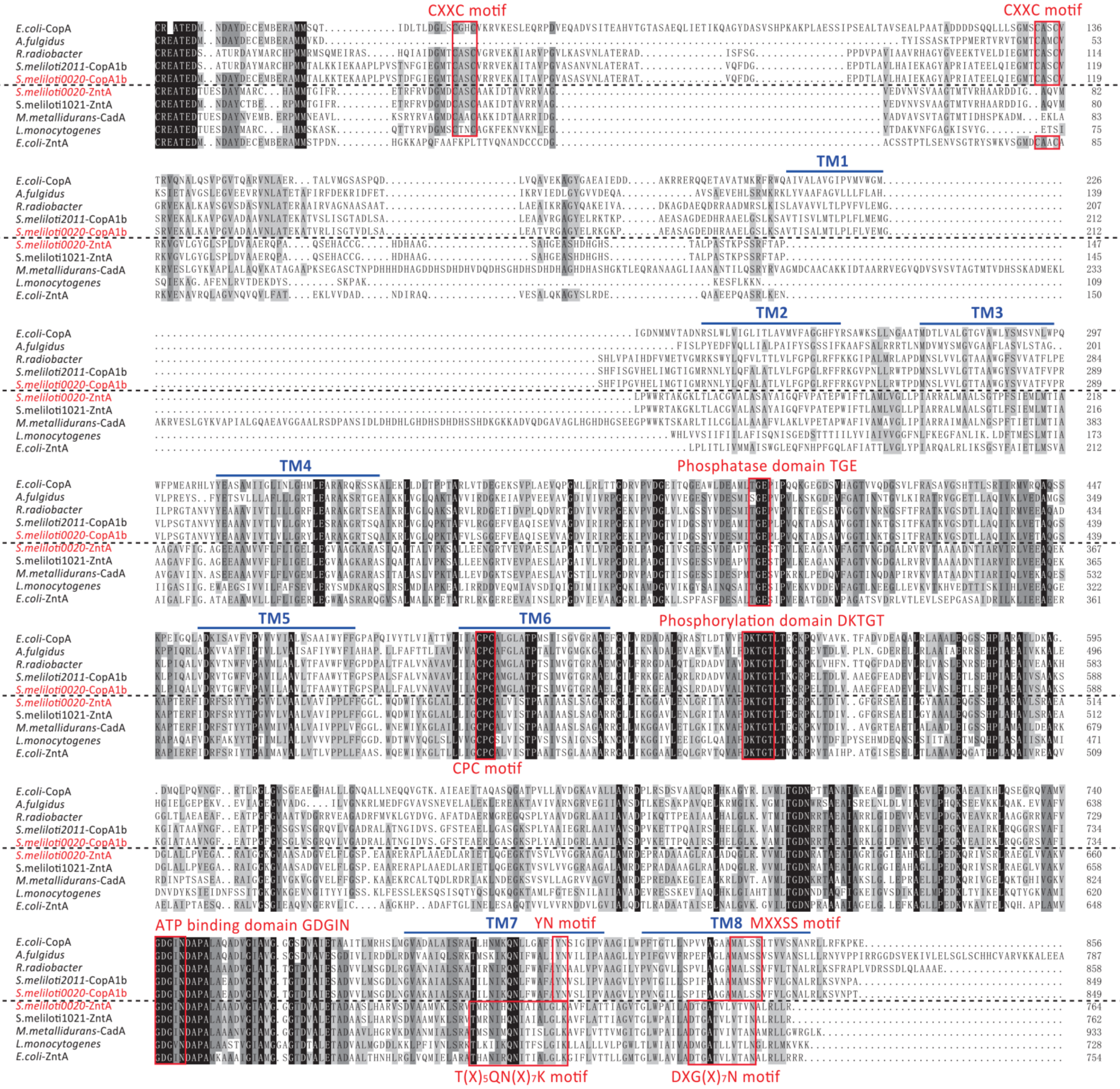

CXXC motif
CXXC motif
E.coli-CopA
A.fulgidus
R.radiobacter
S.meliloti2011-CopA1b
S.meliloti0020-CopA1b
S.meliloti0020-ZntA
S.meliloti1021-ZntA
M.metallidurans-CadA
L.monocytogenes
E.coli-ZntA
TM1
TM2
TM3
TM4
Phosphatase domain TGE
TM5
TM6
Phosphorylation domain DKTGT
CPC motif
ATP binding domain GDGIN
TM7
YN motif
TM8
MXXSS motif
T(X)5QN(X)7K motif
DXG(X)7N motif

Supplementary for Fig. S1 Amino acid sequence alignment of Cu^{+}/Ag^{+}-ATPase (above the dotted line) and Zn^{2+}/Cd^{2+}-ATPase (below the dotted line). Cu^{+}/Ag^{+}-ATPase sequences used for alignment were from *E. coli* (Accession number: Q59385), *A. fulgidus* (Accession number: O29777), *R. radiobacter* (Accession number: A9CJE3), *S. meliloti* 2011 (Accession number: CP004139) and *S. meliloti* CCNWSX0020 (Accession number: EHK77876, this study). Zn^{2+}/Cd^{2+}-ATPase sequences used for alignment were from *E. coli* (Accession number: P37617), *L. monocytogenes* (Accession number: P58414), *M. metallidurans* (Accession number: CCI51008), *S. meliloti* 1021 (Accession number: CAC41511), and *S. meliloti* CCNWSX0020 (Accession number: EHK75591, this study). The conserved domains, metal binding motif, and the signature sequences are boxed and indicated above or below the boxes.

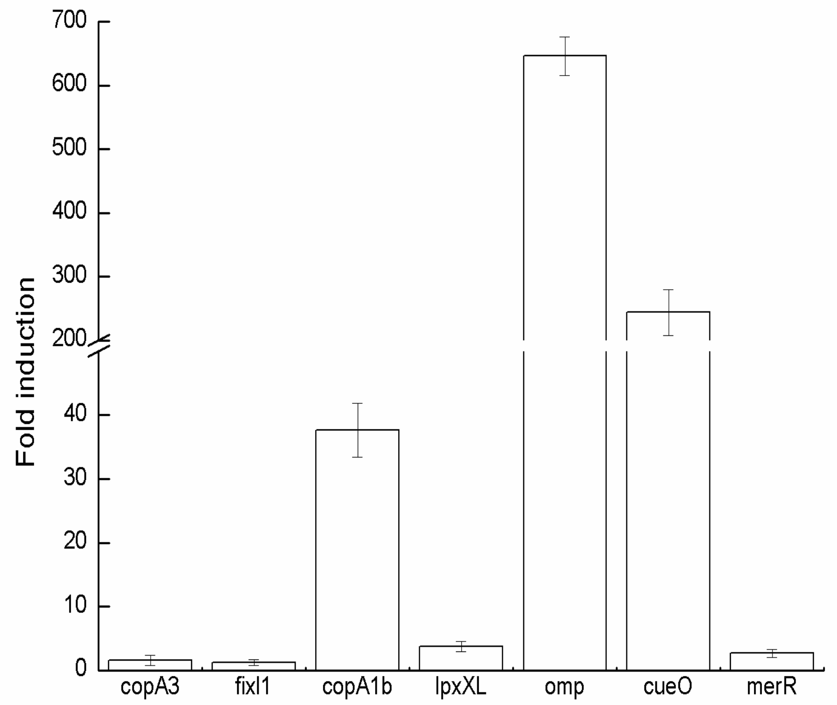

Supplementary for Fig. S2 Expression of genes implicated in copper homeostasis under copper stress. *S. meliloti* CCNWSX0020 wild type strain was incubated with 0.6 mM $CuSO_4$ for 30 min. Samples were then processed for qPCR analysis and normalized against the ribosomal 16S rRNA subunit. Error bars represent standard deviations of three repeats.
